# Direct Adherence Measurement Using an Ingestible Sensor Compared With Self-Reporting in High-Risk Cardiovascular Disease Patients Who Knew They Were Being Measured: A Prospective Intervention

**DOI:** 10.2196/mhealth.6998

**Published:** 2017-06-12

**Authors:** David Thompson, Teresa Mackay, Maria Matthews, Judith Edwards, Nicholas S Peters, Susan B Connolly

**Affiliations:** ^1^ International Centre for Circulatory Health National Heart and Lung Institute Imperial College London London United Kingdom; ^2^ Imperial College Healthcare NHS Trust London United Kingdom; ^3^ National Heart and Lung Institute Imperial College London London United Kingdom

**Keywords:** cardiac prevention and rehabilitation, adherence, mHealth, remote monitoring, cardiovascular diseases, primary prevention, medication adherence, telemedicine

## Abstract

**Background:**

Use of appropriate cardioprotective medication is a cornerstone of cardiovascular disease prevention, but less-than-optimal patient adherence is common. Thus, strategies for improving adherence are recommended to adopt a multifaceted approach.

**Objective:**

The objective of our study was to test a system comprising a biodegradable, ingestible sensor for direct measurement of medication ingestion in a group of patients at elevated cardiovascular risk attending a cardiac prevention and rehabilitation program.

**Methods:**

In this prospective intervention trial in a single group of 21 patients running from April 2014 to June 2015, we measured adherence by self-report and adherence determined objectively by the system. The sensor emits a signal when it encounters the acidic environment of the stomach, detectable by an externally worn patch and linked software app. Longitudinal adherence data in the form of daily progress charts for sensed dosing events as compared with scheduled dosing are visible to patients on their tablet computer’s medication dosing app, thus providing patients with continuous medication adherence feedback. We sought feedback on patient acceptability by questionnaire assessment. Participants used the system for the 12-week period of their cardiac prevention and rehabilitation program.

**Results:**

Only 1 patient at initial assessment and 1 patient at end-of-program assessment reported often missing medication. The remaining patients reported never missing medication or had missing data. Only 12 (57%) of patients overall achieved system-determined adherence of 80% or more, and 3 patients had scores below 40%. Participants reported high levels of acceptability.

**Conclusions:**

This integrated system was well tolerated in a group of 21 patients over an appreciable time frame. Its ability to measure adherence reveals the sizeable disconnect between patient self-reported adherence and actual medication taking and has promising potential for clinical use as a tool to encourage better medication-taking behavior due to its ability to provide continuous patient-level feedback.

## Introduction

Use of appropriate cardioprotective medication is a cornerstone of cardiovascular disease (CVD) prevention, but less-than-optimal patient adherence is common. A meta-analysis and systematic review of 44 prospective studies assessing nearly 2 million participants found that only 60% of patients had good adherence (defined as ≥80%) to CVD medications, and lack of adherence was strongly linked with adverse clinical outcomes [1]. Reasons for nonadherence are multilayered and include patient-, health care provider-, and system-related factors, and thus strategies for improving adherence are recommended to adopt a multifaceted approach [2].

The crucial first step in this process is an accurate assessment of the patient’s medication-taking pattern, not just to make the diagnosis of partial adherence or nonadherence, but also to help resolve ambiguities surrounding drug action (or lack thereof) and side effects, the latter being a key determinant of adherence.

Assessing adherence in daily clinical practice can be challenging. While several different methods exist, ranging from the relatively simple (eg, self-reported adherence derived from patient questionnaires, or assessment of prescription refills or pill counts) to more sophisticated techniques (eg, directly observed therapy, urinary metabolite assays, and electronic monitoring devices that record the frequency and time of the opening of a pill bottle), these measures all have limitations [3]. Self-report in particular is subject to recall bias and social desirability, while indirect methods such as pill counts or analysis of prescription refill data are not synonymous with actual medication taking. Urinary drug metabolite assays have shown some promise in hypertension management but can be confounded by “white coat adherence” and do not necessarily reflect longitudinal medication taking [4,5]. This last limitation similarly applies in directly observed therapy, which is also limited by staff time costs and the potential for tablet concealment [3].

Strategies for reliably measuring and promoting medication adherence in daily clinical practice are thus urgently required. Concomitantly, there is widespread evidence for the increasing use of mobile health technologies in CVD risk reduction and patient self-management [6].

Here we describe our experience of using an innovative telehealth system consisting of an ingestible pill-sensor combination to record an accurate dosing history in patients at elevated risk of CVD attending a CVD prevention and rehabilitation program at our institution. We sought to compare this objective, real-time measurement of adherence with that collected by self-report while also determining the feasibility and acceptability of this technology in everyday clinical practice.

## Methods

### Study Participants and Program Description

The study recruited participants attending the 12-week, nurse-led, multidisciplinary cardiovascular health and rehabilitation program at Imperial College Healthcare NHS Trust, London, UK [7]. Patients were eligible to attend the clinic if they were aged 18 to 80 years and had either established CVD or high multifactorial risk (QRISK2 ≥20%) [8].

The program starts with a detailed initial assessment by each member of the multidisciplinary team, followed by a weekly supervised exercise and educational session and then by an end-of-program assessment. In both assessments, a drug history is recorded and standard questions regarding medication adherence are posed. A key tenet of the program is medical risk factor modification and prescription of appropriate cardioprotective medication at evidence-based doses. Education is provided regarding medication and its indication to promote drug adherence.

We invited consecutive patients attending the baseline assessment from April 2014 to June 2015 to participate in our study. In addition to the criteria for attendance at the clinic, patient enrollment required active use of CVD medications, and we excluded patients due to (1) lack of fluency in English, (2) literacy problems, (3) pill swallowing difficulties, or (4) psychological ill health sufficient to affect study involvement.

### Intervention

**Figure 1:**
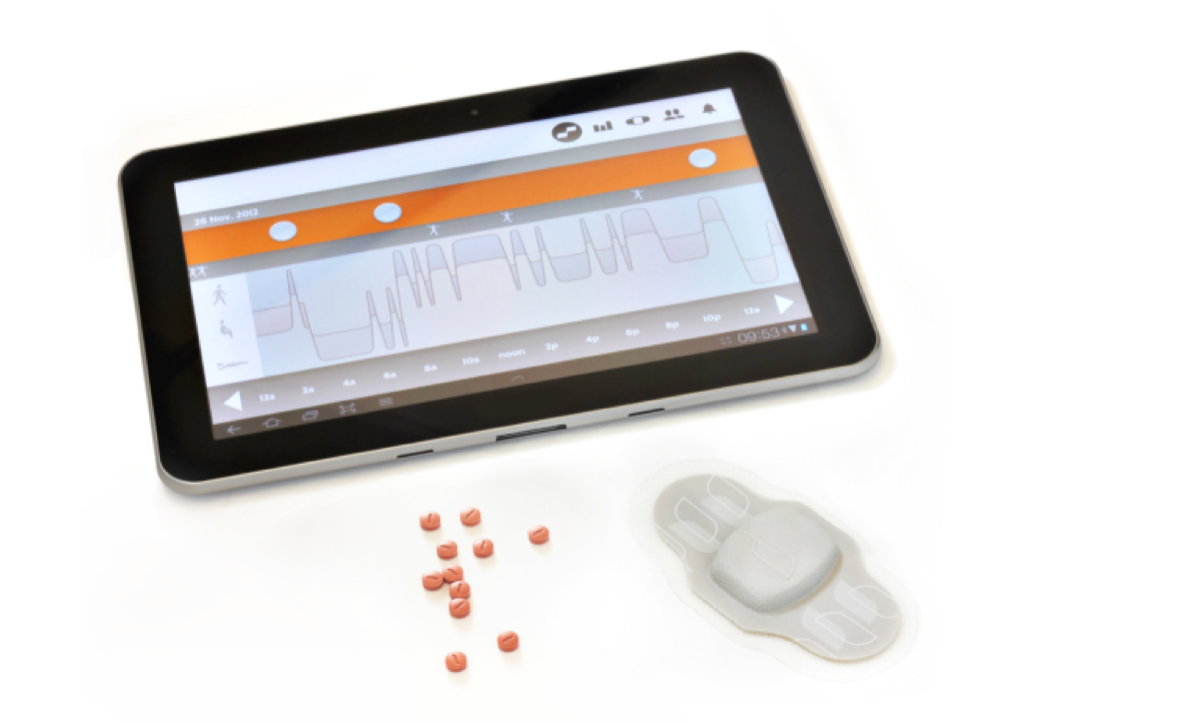
Integrated Lifenote system featuring a tablet computer, ingestible sensor, and externally worn patch.

After we obtained informed consent, we gave study participants instructions during an education session (“on-boarding”) on how to use the Lifenote system (Proteus Digital Health, Inc, Redwood City, CA, USA). The system requires the user to ingest a biodegradable sensor (shaped like a small pill) alongside each scheduled medication dosing (Figure 1).

**Figure 2:**
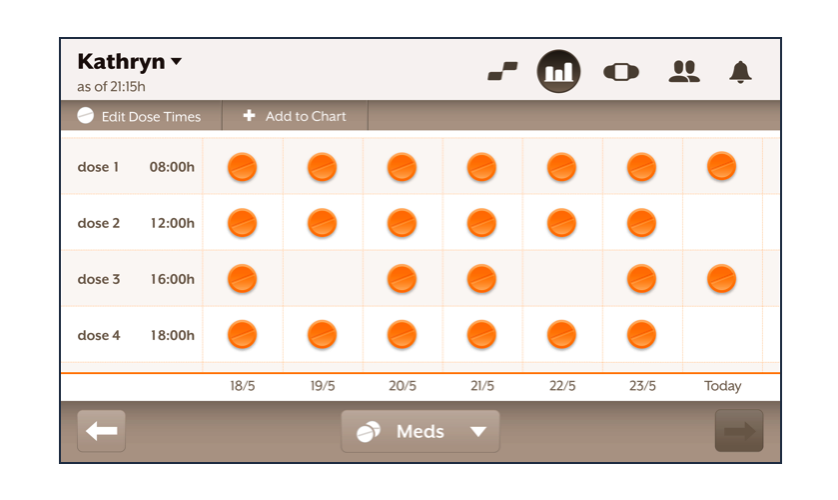
On-screen representation of the device’s scheduled dosing table. Each sensed dosing event is represented by an orange pill in each cell. Columns correspond to days of the week and rows to each scheduled dosing event for that day.

The sensor then emits a signal when it encounters the acidic environment of the stomach, which is detectable externally by a patch worn over the left upper quadrant of the abdomen. The patch then sends a signal via Bluetooth technology to a paired tablet computer or mobile phone loaded with the system’s software app. The patches are changed daily and have inbuilt sensors to validate patch application. The system has a positive detection rate of 97% using directly observed ingestion as a reference standard for comparison [9]. At the on-boarding session, scheduled doses for each day of the week were entered according to that patient’s prescription, and each successful ingestion was registered as an event on that patient’s progress chart within the software app that matched expected doses with sensed dosing events (Figure 2). Patients had access to telephone technical support to troubleshoot connectivity issues. With regard to medication taking, they received an on-screen notification if 30 minutes had passed without successful registration of a sensed dosing event.

### Outcome Measures

#### Adherence

##### System-Determined Adherence

We defined adherence as the total number of successful ingestions detected by the patch, expressed as a percentage of the total number of planned ingestion events for that period. We did not apply a time restriction. We excluded periods when a patient was not wearing a patch. A minimum 7-day period of valid data was required to be included in the data analysis.

##### Adherence by Self-Report

Patients were routinely asked as part of their initial and end-of-program assessments if they “missed taking or altered the dose of your prescribed medicine.” The permitted responses were “never,” “seldom,” “often,” “always,” and “not applicable.”

#### Acceptability

We assessed acceptability of the system to patients using a questionnaire at the end-of-program assessment.

### Data and Statistics

For this study, we extracted sensed adherence data from the system. We then collated descriptive statistics in Stata version 14.1 for Mac (StataCorp LP) and Excel for Mac version 15 (2015; Microsoft Corporation). Data are presented as mean (SD) or, in the case of skewed data, median and range from minimum to maximum. Percentage adherence has been rounded to whole numbers.

## Results

**Figure 3:**
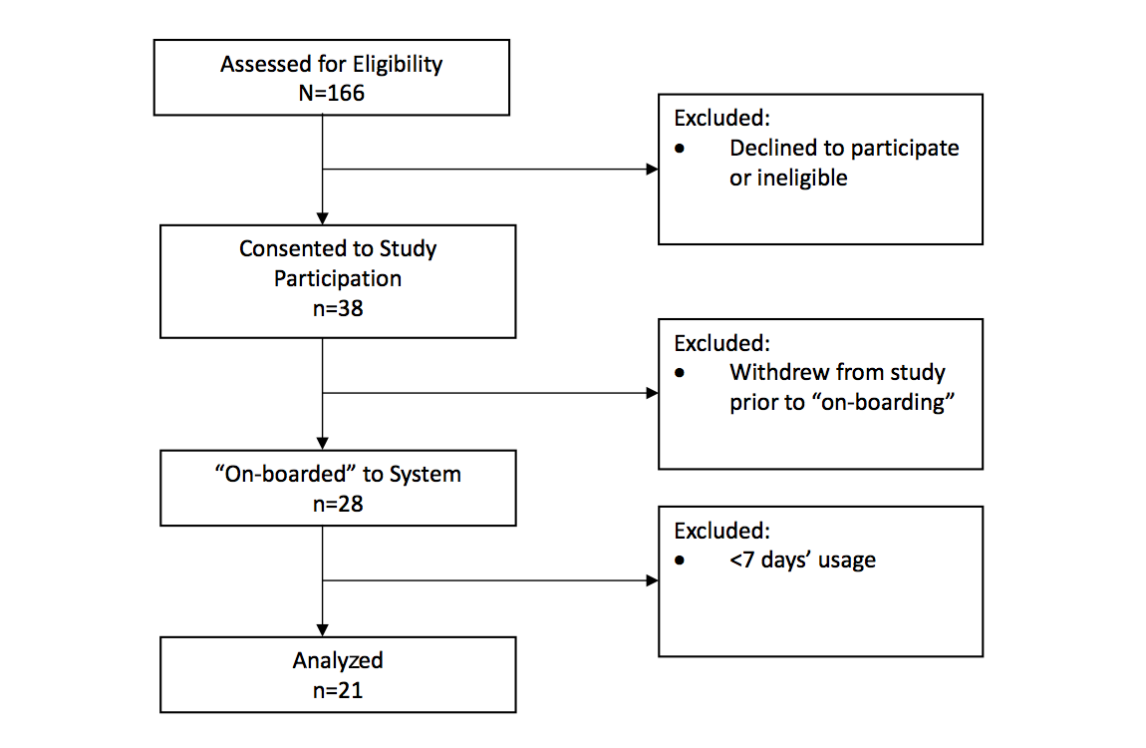
Patient flowchart.

We invited 166 consecutive patients who met study eligibility criteria to participate (Figure 3), of whom 38 (22.9%) agreed and provided written, informed consent. Of those, 10 patients withdrew prior to starting to use the system, leaving a remaining 28 participants with use experience. A total of 7 participants ended their use period prematurely for reasons including the following: “Went away on holiday,” “preferred app on phone.” “didn’t want to use the device,” “device wasn’t for me,” and “off-boarded due to adverse event.”

Thus, 21 patients with a minimum of 7 days of use experience were the focus of our analysis. Table 1 outlines their baseline characteristics. Median patch wear sensor time for the group, expressed in days of collected data as a percentage of the total number of days where data were expected, was 91%, (range 49%-100%). Patients had a mean age of 62 (SD 12) years, and the majority were male (n=15, 71%) with a mean body mass index of 30.7 (SD 5.0) kg/m^2^. A total of 14 (67%) were primary prevention patients (increased CVD risk, type 2 diabetes mellitus), and the remainder were enrolled for secondary prevention (n=7, 33%). Most patients were taking 2 or more CVD drugs at their initial assessment (Table 1); 3 patients were taking a single CVD drug, and 2 patients enrolled with a view to commencing statin therapy during the program but did not start and instead remained on at least one non-CVD drug throughout.

**Table 1:** Baseline patient characteristics (n=21).

Characteristic		Mean (SD) or n (%)
Age in years, mean (SD)		62 (12)
Male, n (%)		15 (71)
Body mass index in kg/m^2^, mean (SD)		30.7 (5.0)
**Race/ethnicity, n (%)**		
	White	15 (71)
	Other	4 (19)
	Asian	1 (5)
	Black	1 (5)
**Diagnosis, n (%)**		
	Primary prevention	14 (67)
	Secondary prevention	7 (33)
**Cardiovascular drugs prescribed per patient^a^, n (%)**		
	0	2 (10)
	1	3 (14)
	2	9 (43)
	3	3 (14)
	4	3 (14)
	5	1 (5)

^a^Antiplatelet agents, statins, fibrates, other lipid-lowering drugs, angiotensin converting enzyme inhibitors, angiotensin receptor blockers, β-blockers, calcium channel blockers, diuretics.

**Figure 4:**
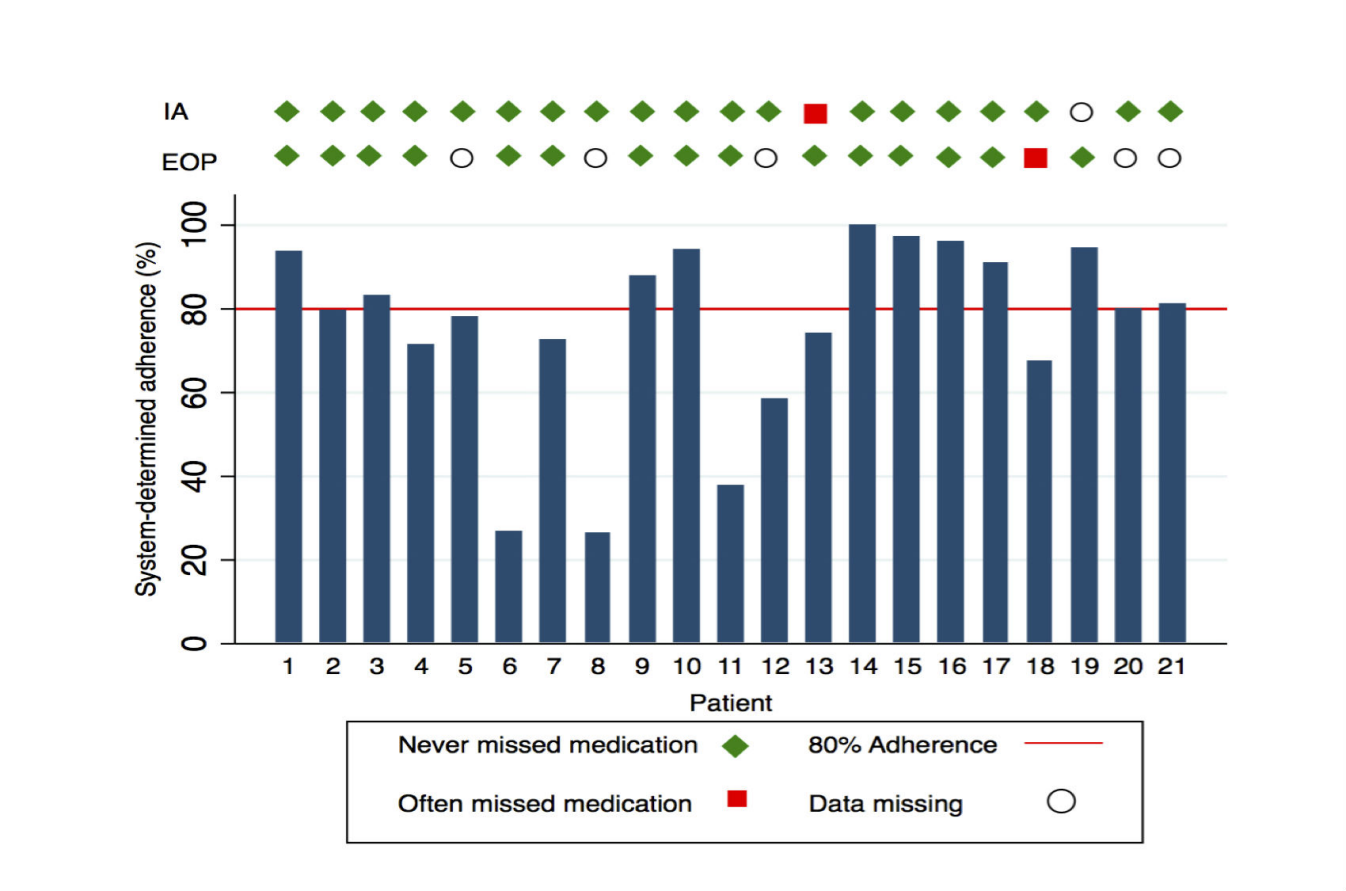
System-determined adherence (%) by participant. Markers indicate self-reported adherence responses at initial assessment (IA) and end-of-program (EOP) assessment.

### Self-Reported Adherence

Of the 21 patients, 19 (90%) reported that they never missed medication at baseline and 1 patient reported that they often missed medication (Figure 4). Data were missing for 1 participant at the initial assessment. None of the patients reported seldom missing medications, and none stated that the question was not applicable.

Of the 19 participants who reported never missing medication at the initial assessment, 13 reported never missing medication at the end-of-program assessment (Figure 4). One patient reported never missing medication at their initial assessment and then reported often missing their medication at the end-of-program assessment. One patient who reported at their initial assessment that they often missed their medication reported that they never missed it at their end-of-program assessment. Data were not available on 5 participants at the end-of-program assessment due to loss to follow-up.

### System-Determined Adherence

Overall, the proportion achieving good adherence (defined as ≥80%) was 12 of 21 (57%) (Figure 4). The median system-determined adherence was 80%, but there was substantial interindividual variability (26%-100%). Patient 13, who had reported poor adherence at their initial assessment but good adherence at their end-of-program assessment, had a measured adherence of 74%. Conversely, patient 18, who reported good adherence at their initial assessment but poor adherence at their end-of-program assessment, had a measured adherence of 67%.

### Acceptability

A total of 8 (38%) patients agreed or strongly agreed with the statement that “the patch was comfortable,” while 5 (24%) were neutral and a minority disagreed (Table 2). The majority of the patients 14 (67%) found the system easy to use. Overall, 11 (52%) felt that they were less likely to miss doses using the system, with approximately two-thirds responding that they would continue to use the system in the future, although one-third indicated that they would require amendments before doing so (Table 2).

A single participant experienced an adverse event due to patch-related contact dermatitis.

**Table 2:** Number (%) of participants’ choosing each possible response to questionnaire items regarding acceptability (n=21).

Response	Q1^a^	Q2^b^	Q3^c^	Q4^d^
Strongly disagree	0	0	1 (5)	
Disagree	2 (9)	0	1 (5)	
Neither or neutral	6 (29)	5 (24)	9 (43)	
Agree	7 (33)	9 (43)	5 (24)	
Strongly agree	4 (19)	5 (24)	3 (14)	
No response	2 (10)	2 (9)	2 (9)	
I would continue to use the system				7 (33)
I would use the system if changes were made				4 (19)
I would not use the system				6 (29)
No response				4 (19)

^a^“Using Lifenote meant I was less likely to miss taking my tablets.”

^b^“Lifenote was easy for me to use.”

^c^“The patch was comfortable.”

^d^Desire to continue system use.

## Discussion

To the best of our knowledge, this is the first inpatient study using a system featuring an ingestible, biodegradable sensor for the objective assessment of adherence in patients attending a cardiac prevention and rehabilitation program. Self-reported adherence was high at the baseline assessment, with 90% reporting that they never missed medication. However, adherence measured by sensing of events demonstrated that only 57% achieved good adherence (defined as ≥80%) over the course of the program. This is consistent with the literature, where it is well recognized that measurement of adherence by self-report leads to overreporting, in turn due to a combination of factors, including recall bias and social desirability.

What is intriguing is that, despite the fact that participants knew their ingestion event record would be scrutinized, only 1 of the 19 who reported good adherence at baseline changed their reporting of adherence from “good” to “bad” at the end-of-program assessment, while those (n=3) with the worst adherence (<40%) continued to report good adherence at *both* time points. These data, therefore, underscore not only the lack of reliability of self-report as a measure of adherence, but also the psychological complexities of medication-taking behavior.

The only patient who admitted to poor adherence at the initial assessment reported never missing medication at the end-of-program assessment and achieved a sensed adherence of 74%. Although somewhat subjective, this may perhaps represent a positive change in behavior. Indeed, the majority of participants also felt that the system helped them to avoid missing doses. While some data were missing at the end-of-program assessment due to loss to follow-up, the rate of follow-up attendance in this study compares favorably with the expected typical follow-up attendance rate of approximately 60% for such a program.

The device was well tolerated, with only 1 adverse event (an episode of patch-related contact dermatitis), which is a well-recognized side effect. Despite this being an older population, the majority found the system easy to use. Recent studies in patients with hypertension or diabetes of similar average age also found high levels of acceptability and low levels of adverse events [10,11].

This high level of acceptance likely reflects the permeation of mobile phone or tablet technology into society as a whole. The evidence for mobile health interventions (mHealth) overall is steadily increasing, and one of the most prolific areas of development is CVD risk reduction [6]. Simple interventions such as text messaging have proven effective in improving clinical outcomes in a secondary prevention population, and the same research group is now studying the effect of text messages on medication adherence [12,13].

The main potential of this technology, therefore, relates to not only measurement of adherence reliably but also a strategy to increase adherence. Access to real-time adherence data and progress logs could also be extended to a patient’s family or medical team, allowing them to play a much more active role in the patient’s medication management and potentially to overcome other common barriers to health care such as limited mobility or distance from care givers or the health care setting [14]. These concepts, however, would need to be tested in the context of well-designed, controlled studies including a determination of cost effectiveness before being put into widespread use.

There are also key limitations to our study that need to be addressed. First, patients choosing to participate in the study were clearly a highly selected group (approximately 20% of those invited), a majority of whom were white, and the results, therefore, may not be reflective of the general population. We expect, however, that the high degree of self-selection would have resulted in higher measured adherence, as those who were more motivated and had good medication behavior were more likely to enter the study.

Second, the duration of the period studied was relatively short (about 3 months) and may not necessarily reflect medication adherence in the longer term.

Third, to register a dosing event, the ingestible sensors had to be coingested with the patient’s own medications, and it is entirely possible that participants could have ingested the medication alone, without ingesting the sensor (leading to underestimation of ingested events), or the sensor alone, without the accompanying medication (leading to an overestimation of adherence). Sensors that are coformulated or overencapsulated with prescribed medicines would overcome this issue, but this would also add to the complexity and cost of the intervention.

Fourth, we did not have a control group for comparison and therefore any benefits seen cannot be separated from those resulting from participation in cardiac prevention and rehabilitation alone.

In conclusion, this integrated telehealth system using an ingestible pill sensor demonstrated lower levels of adherence to CVD medications than that indicated by self-report. The technology was both safe and acceptable to patients. Larger studies are needed to determine the system’s potential for measuring and promoting adherence on a wider scale.

## Abbreviations

CVD: cardiovascular disease
