# Metadata Correction: Direct Adherence Measurement Using an Ingestible Sensor Compared With Self-Reporting in High-Risk Cardiovascular Disease Patients Who Knew They Were Being Measured: Prospective Intervention

**DOI:** 10.2196/mhealth.8317

**Published:** 2018-04-27

**Authors:** David Thompson, Teresa Mackay, Maria Matthews, Judith Edwards, Nicholas S Peters, Susan B Connolly

**Affiliations:** ^1^ International Centre for Circulatory Health National Heart and Lung Institute Imperial College London London United Kingdom; ^2^ Imperial College Healthcare NHS Trust London United Kingdom; ^3^ National Heart and Lung Institute Imperial College London London United Kingdom

In the paper by David Thompson et al, “Direct Adherence Measurement Using an Ingestible Sensor Compared With Self-Reporting in High-Risk Cardiovascular Disease Patients Who Knew They Were Being Measured: A Prospective Intervention” (JMIR Mhealth Uhealth 2017;5(6):e76), author Nicholas S Peters's middle name initial was omitted. 

The author's name has been corrected and the corrected article will appear in the online version of the paper on the JMIR website on April 27, 2018, together with the publication of this correction notice. Because this was made after submission to PubMed, Pubmed Central, and other full-text repositories, the corrected article also has been re-submitted to those repositories.

